# Preliminary reference range for B cell subpopulations in peripheral blood of healthy Malaysian children aged 2–15 years

**DOI:** 10.1038/s41598-026-40720-2

**Published:** 2026-03-02

**Authors:** Jalilah Jamaluddin, Intan Hakimah Ismail, Mohd Azri Zainal Abidin, Siti Mardhiana Mohamad, Hasni Mahayidin

**Affiliations:** 1 Clinical Immunology Unit, Department of Paediatrics, Faculty of Medicine and Health Sciences, Universiti Putra Malaysia, Kuala Lumpur, Malaysia; 2 Department of Community Health, Advanced Medical and Dental Institute, Universiti Sains Malaysia, Bertam, Pulau Pinang Malaysia; 3 Department of Pathology, Faculty of Medicine and Health Sciences, Universiti Putra Malaysia, Serdang, Selangor Malaysia

**Keywords:** Humoral immunity, B cells, class-switched memory B cells, Age-specific reference range, Diseases, Immunology

## Abstract

Physicians rely on reference values from healthy populations to guide clinical decisions regarding B-cell subpopulations in primary immunodeficiency. While age-dependent reference ranges have been reported in several populations, no study has established these values for Malaysian children. Given that B-cell subpopulation distributions may vary between populations, we aimed to define reference ranges for total B cells, transitional B cells, naïve B cells, total memory B cells, switched and non-switched memory B cells, and plasmablasts in Malaysian children aged 2 to 15 years. Blood samples taken from 85 children aged 2 to 15 years were evaluated for the distribution of B cell subsets. Absolute numbers and percentages were determined for total B cells (CD19^+^), transitional B cells (CD19^+^CD27^−^CD24^+ bright^CD38^+bright^), naïve B cells (CD19^+^CD27^−^IgD^+^), total memory B cells (CD19^+^CD27^+^), class-switched memory B cells (CD19^+^CD27^+^IgM^−^IgD^−^), non-classical switched memory B cells(CD19^+^CD27^−^IgM^−^IgD^−^), non-switched memory B cells (CD19^+^CD27^+^IgM^+^IgD^+^), and plasmablasts (CD19^+^CD27^+^CD38^+ bright^). We observed age-dependent variations in most B-cell subpopulations, with naïve B cells being predominant, followed by memory B cells, while plasmablasts were present in trace amounts across all ages. Additionally, certain of B-cell subpopulations (total memory B cells and class-switched memory B cells) were observed at higher frequencies in female children compared to males. This study provides age-specific reference values for B cell subsets in a paediatric population, which may serve as a valuable guideline for diagnosing children with suspected immunodeficiency.

## Introduction

B cells are essential components of the immune system, playing a crucial role in adaptive immunity. The development of B cells involves a complex maturation process that begins with hematopoietic stem cells (HSCs) in the bone marrow, progressing through various stages from immature B cells to transitional B cells and finally into mature naïve B cells^1,2^. Immature B cells exit the bone marrow and enter the peripheral circulation as transitional B cells (CD19^+^CD38^++^CD24^++^ or CD19^+^CD27^−^IgD^+^CD38^+^), which further mature into naïve B cells (CD19^+^IgD^+^CD27^−^). Upon antigen recognition, naïve B cells proliferate and differentiate into short-lived plasmablasts (CD19^+^CD138^++^ or CD19^+^CD27^+^CD38^++^), plasma cells (CD38^+^CD138^+^) or memory B cells (CD19^+^CD27^+^) within the germinal centre^3^. Memory B cells provide long-term protective immunity and can be further subclassified into class-switched memory B cells (CD19⁺CD27⁺IgM⁻IgD⁻), non-switched memory B cells, also known as marginal zone B cells (CD19⁺CD27⁺IgM⁺IgD⁺), IgM-only memory B cells (CD19⁺CD27⁺IgM⁺IgD⁻) and IgD-only memory B cells (CD19⁺CD27⁺IgM⁻IgD⁺)^3–6^.

Abnormal B cell distribution has been linked to primary immunodeficiencies, recently termed inborn errors of immunity. The International Union of Immunological Societies (IUIS) has recognised a reduced number of memory B cells as a hallmark of combined immunodeficiency, hyper-IgE syndrome, and activated PI3K-delta syndrome (APDS). Additionally, decreased plasmablast levels have been used to assess immune dysregulation, particularly in diagnosing NFAT5 haploinsufficiency. Transitional B cells are also measured in patients suspected of having common variable immunodeficiency or combined immunodeficiency. Although B cell subpopulation analysis is crucial for investigating primary immunodeficiencies (PID), reference ranges for B cell distribution remain limited, making comparison with patient populations challenging^7^.

Establishing a reference range for B cell subpopulations is crucial for guiding physicians in diagnosing young patients with suspected PID. However, these reference values may vary across laboratories due to differences in population demographics, geographical areas, selection criteria for healthy subjects, and sample preparation techniques, all of which contribute to the wide range of normal values reported^8–10^. These variations can introduce biases, especially in immunodeficiency assessments. Therefore, this study quantified and analysed B-cell subpopulations in children aged 2 years to 15 years to establish age-dependent reference intervals for humoral immune parameters, assess age-related variations in B-cell subpopulations, and explore differences between genders. To the best of our knowledge, this is the first study to define the normal distribution of B-cell subpopulations in peripheral blood in Malaysia, specifically at our centre, the Clinical Immunology Centre, Universiti Putra Malaysia.

## Materials and methods

### Study population of the healthy controls

A total of 85 healthy individuals aged 2 to 15 years were voluntarily enrolled in this study between November 2022 and December 2023. Participants were referred to the paediatric clinic at Hospital Sultan Abdul Aziz Shah, Universiti Putra Malaysia, for developmental assessments and had received standard childhood immunisations according to the Malaysian National Immunisation Programme. Children with immune disorders, chronic diseases, syndromic conditions confirmed by geneticists, those receiving intravenous immunoglobulin treatment, or those on immunosuppressive medications were excluded. To minimise the potential impact of acute immune activation on B-cell subset distribution, only children who were clinically well and infection-free at the time of blood sampling were included in the study.

### Sample preparation and data collection

**Fig. 1:**
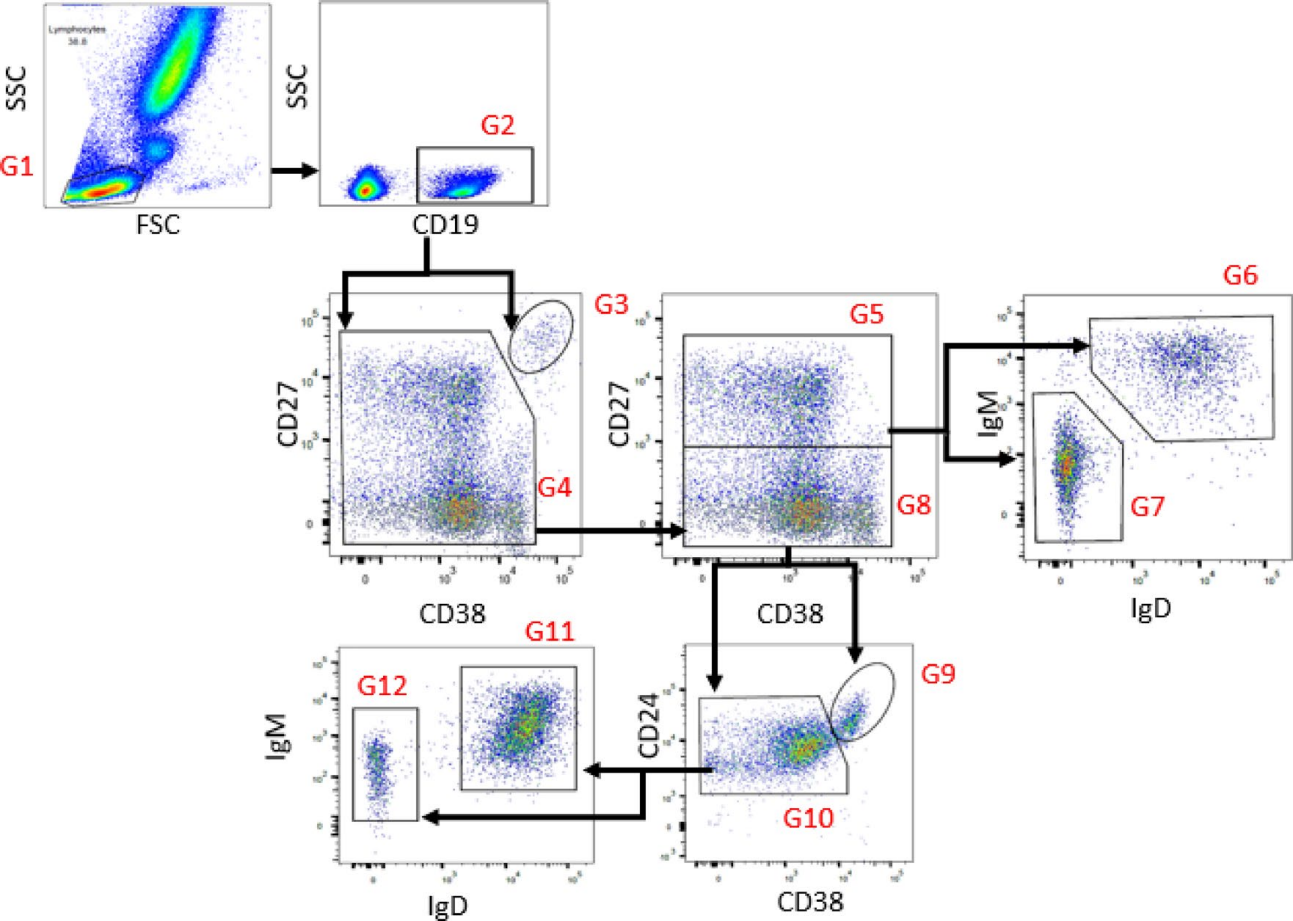
Gating strategies used for the determination of B-cell subpopulations. The lymphocyte population was identified based on side-scatter (SSC) and forward-scatter (FSC) characteristics (G1). After gating on CD19^+^ B cells (G2), the B-cells were further analysed based on the expression of CD27 and CD38, CD24 and CD38 or IgD and IgM. The following B-cell subpopulations have been identified in this study: plasmablast (CD27^+^CD38^+bright^, G3), total memory B cells (CD27^+^, G5) after gating on CD27^−/+^ CD38^−/+^ (G4), non-switched memory B cells (IgD^+^IgM^+^, G6) and class-switched memory B cells (IgD^−^IgM^−^, G7) after gating on CD27^+^ (G5), transitional B cells (CD24^+ bright^CD38^+bright^, G9) after gating on CD27^−^ (G8), naïve B cells (IgD^+^IgM^+^, G11) and non-classical switched memory B cells (IgD^−^IgM^−^, G12) after gating on CD38^−/+^CD24^−/+^ (G10).

Blood samples were collected in ethylenediamine tetra-acetic acid (EDTA) tubes and processed within 24 h. Flow cytometric analysis was performed using FACSLyrics (BD Biosciences, New Jersey, United States) with six-colour immunostaining for the following markers and fluorochromes: CD19-PerCPCy5.5, CD27-APC, CD38-PE-Cy7, CD24-APC-H7, IgD-PE, and IgM-BB515. Data generated were analysed using FlowJo software (BD Biosciences, New Jersey, United States). Briefly, whole blood was washed twice with stain buffer, incubated with immunofluorescence staining, and treated with FACSLysing solution (BD Biosciences, New Jersey, United States). The gating strategies are described in Fig. 1. The absolute number of cells was calculated by multiplying the relative proportion (percentage) of B-cell subpopulations by the absolute number of B cells obtained from T cell, B cell and natural killer cell (TBNK) enumeration or the lymphocyte count from a complete blood count (CBC) performed on the same day. We then identified each B-cell subsets based on the following markers: total B cells (CD19^+^), transitional B cells (CD19^+^CD27^−^CD24^+ bright^CD38^+bright^), naïve B cells (CD19^+^CD27^−^IgD^+^), total memory B cells (CD19^+^CD27^+^), switched memory B cells (CD19^+^CD27^+^IgM^−^IgD^−^), non-classical switched memory B cells(CD19^+^CD27^−^IgM^−^IgD^−^), non-switched memory B cells (CD19^+^CD27^+^IgM^+^IgD^+^), and plasmablasts (CD19^+^CD27^+^CD38^+ bright^). As gating was restricted to CD19⁺ cells, mature plasma cells (typically CD19⁻CD138⁺) were not assessed in this study, and the CD19⁺CD27⁺CD38⁺^bright^ population was classified as circulating plasmablasts.

### Statistical analysis

Data were analysed using SPSS version 27 and Microsoft Excel^®^. Reference values were established for three age groups ranging from 2 to 15 years. These reference values were determined based on the 5th and 95th percentiles, representing the lower and upper limits, respectively. Medians and interquartile ranges were calculated for each age group. For statistical comparisons, Mann-Whitney test was used to analyse differences in B-cell subpopulations between gender, Kruskal Wallis test was performed to assess differences in B-cell subpopulations among age groups, while Spearman’s rank correlation test was used to evaluate age-dependent changes in B cell-subpopulations. A p-value < 0.05 was considered statistically significant.

## Results

Initially, 82 healthy children aged 2 to 15 years old were recruited; however, seven were excluded as outliers based on Tukey’s and Mahalanobis tests. B-cell subpopulations were analysed in three age groups: 2 to 4 years (*n* = 27), 5 to 9 years (*n* = 31), and 10 to 15 years (*n* = 17). Demographic details are provided in Table 1. TBNK enumeration was performed for all participants at the time of recruitment as part of routine immunological assessment. All subjects had normal absolute lymphocyte counts based on established reference values^11^. Although enrolment screening was guided by established paediatric reference values, all lymphocyte subset distributions observed in this cohort were reviewed and confirmed to be within age-appropriate ranges based on the data generated during the study.

**Table 1:** Demographic details for the 75 healthy children tested according to the age group.

Age group	Median age	Total number of subjects	Gender (Male/Female)
2 to 4 years old	4.081	27	14/13
5 to 9 years old	7.973	31	15/16
10 to 15 years old	11.682	17	11/6
Total	6.553	75	75

The composition of B-cell subpopulations exhibited age-related variations. The total B cell frequency showed a decreasing pattern across the age groups where the median decreased from 22.8% in 2- to 4-year-old group to 18.6% in 10- to 15-year-old group. In contrast, transitional B cells, total memory B cells, non-switched memory B cells, class-switched memory B cells, non-classical switched memory B cells, and plasmablast cells displayed an initial increase from the 2- to 4-year-old group to the 5- to 9-year-old group, followed by a decline in the 10- to 15-year-old group with significant differences were observed in the total memory B cell frequency (*p* < 0.001) and in class-switched memory B cells frequency (*p* < 0.001). In addition, the frequency of naïve B cells gradually increased throughout the age group from 81.5% in 2- to 4-year-old group to 83.6% in 5- to 9-year-old group and subsequently to 84.7% in 10- to 15-year-old group. However, no significant differences in frequency were detected across age groups for total B cells, transitional B cells, naïve B cells, non-switched memory B cells, or plasmablasts.

**Fig. 2:**
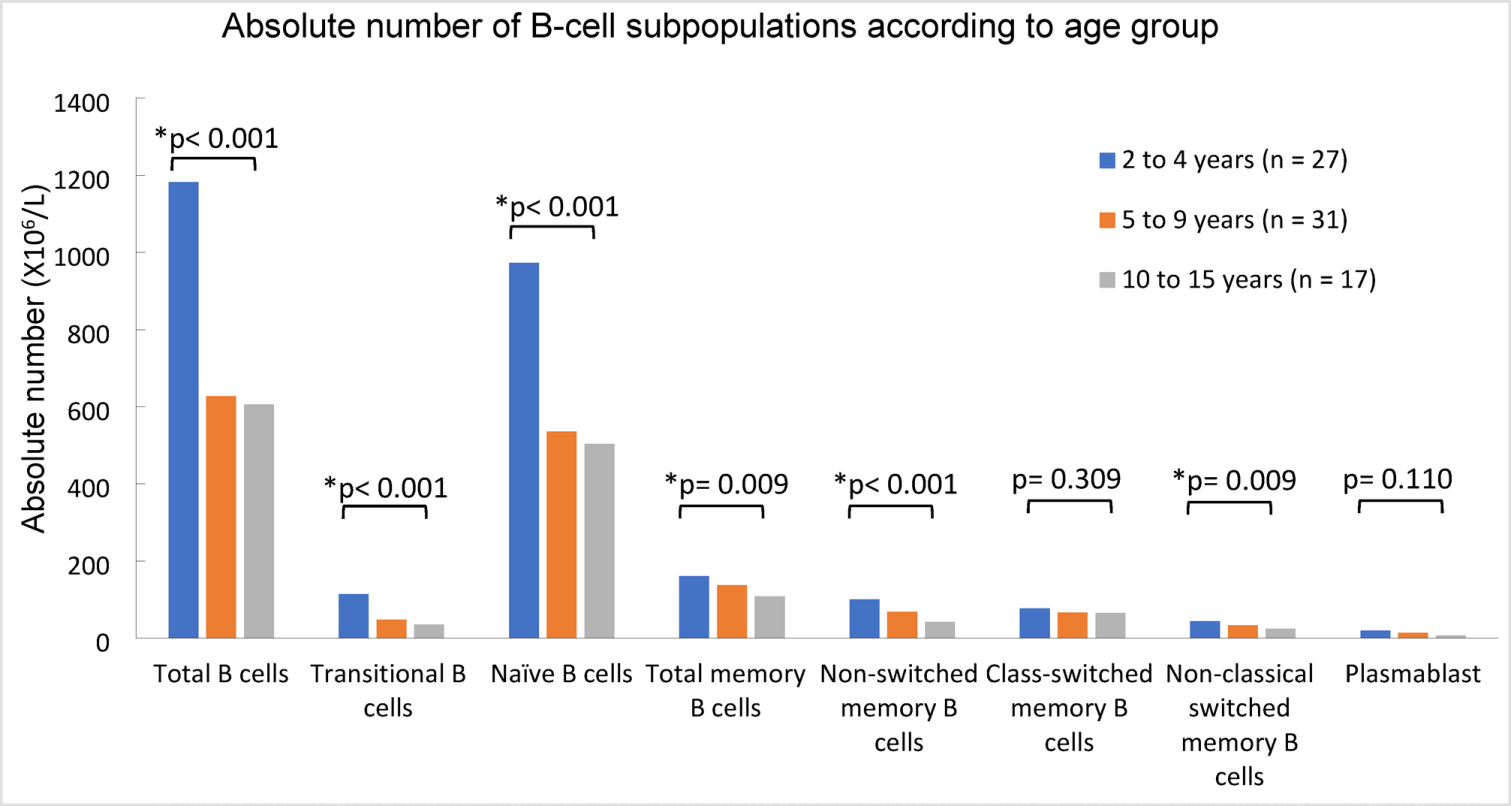
Absolute numbers of B-cell subpopulations in healthy Malaysian children aged 2–15 years. The distribution of absolute number of B-cell subpopulations in healthy Malaysian children including total B cells, transitional B cells, naïve B cells, total memory B cells, non-switched memory B cells, switched memory B cells, non-classical switched memory B cells and plasmablasts. Comparisons are shown across three age groups: 2–4 years, 5–9 years, and 10–15 years. *Indicates significant differences, *p* < 0.05.

The absolute number of each B-cell subpopulation also showed age-related changes. In total B cells, the absolute number was declined as individuals aged, with significant differences observed between the 2- to 4-year-old group and the 5- to 9-year-old group (*p* < 0.001), as well as between the 2- to 4-year-old group and the 10- to 15-year-old group (*p* < 0.001). A similar pattern with significance differences were identified in the transitional B cells, naïve B cell, total memory B cells, non-switched memory B cells, and non-classical switched memory B cells. The absolute number of transitional B cells and naïve B cells also showed significant decline in both subset populations between the 2- to 4-year-old group and 5- to 9-year-old group (*p* < 0.001), as well as between 2- to 4-year-old group and the 10- to 15-year-old group (*p* < 0.001). In the case of total memory B cells, the absolute number was significantly lower in the 10- to 15-year-old age group compared to the 2- to 4-year-old group (*p* = 0.002). In addition, the absolute number of non-switched memory B cells and non-classical switched memory B cells also demonstrated significant differences whereby lower absolute number was observed in 10- to 15-year-old group when compared to 2- to 4-year-old group (*p* < 0.001 and *p* = 0.007, respectively). Conversely, both class-switched memory B cells and plasmablasts exhibited declining in absolute number from 2 to 4 to 10- to 15-year-old group, although these changes were not statistically significant. The absolute numbers of each B-cell subpopulation are illustrated in Fig. 2. The median values and interquartile ranges (5th and 95th percentiles) for the frequencies and absolute numbers of B cell subpopulations across age groups are provided in Tables 2 and 3.

**Table 2:** Frequencies for each B cell subpopulation in distinct age groups.

Age group	Total B	Transitional B	Naïve B	Total memory B	Non-switched memory B	Class- switched memory B	Non-classical switched memory B	Plasmablast
2 to 4 years (*n* = 27)	22.8 (11.8–31.7)	8.8 (1.9–23.9)	81.5 (68.0–93.1)	14.2 (7.8–28.2)	8.5 (4.3–16.2)	6.2 (2.3–12.7)	3.29 (1.22–11.95)	1.64 (0.17–10.12)
5 to 9 years (*n* = 31)	19.0 (12.1–30.1)	7.7 (0.7–19.4)	83.6 (68.8–91.5)	19.8 (10.5–35.7)	10.4 (4.5–15.3)	10.3 (3.8–20.4)	4.91 (1.43–14.08)	1.92 (0.59–8.25)
10 to 15 years (*n* = 17)	18.6 (10.1–31.4)	7.1 (0.6–10.8)	84.7 (76.3–92.2)	17.9 (11.7–33.5)	7.7 (4.8–12.2)	9.1 (5.9–20.1)	3.33 (1.51–10.80)	1.26 (0.27–13.80)

All results are presented as median values with ranges between 5th and 95th percentile.

**Table 3:** Absolute number for each B cell subpopulation in distinct age groups.

Age group	Total B cells	Transitional B	Naïve B	Total memory B	Non-switched memory B	Class-switched memory B	Non- classical switched memory B	Plasmablast
2 to 4 years (*n* = 27)	1182.1 (614.3–1975.4)	113.5 (15.6–241.6)	973.3 (487.9–1714.9)	160.8 (77.9–452.6)	100.8 (40.4–243.3)	76.7 (23.6–204.1)	43.7 (11.5–169.6)	19.7 (1.7–117.0)
5 to 9 years (*n* = 31)	627.0 (353.8–1658.6)	47.6 (7.2–213.2)	535.8 (309.0–1337.5)	137.3 (52.9–384.2)	67.7 (27.8–169.4)	66.5 (22.2–207.2)	33.0 (8.5–86.8)	13.8 (4.4–45.6)
10 to 15 years (*n* = 17)	606.0 (226.0–1130.0)	34.7 (2.5–86.9)	503.6 (205.2–956.0)	107.9 (46.6–241.2)	41.9 (21.4–87.8)	65.2 (22.7–144.7)	24.5 (3.8–77.8)	6.6 (1.4–106.7)

All results are presented as median values with ranges between 5th and 95th percentile.

**Fig. 3:**
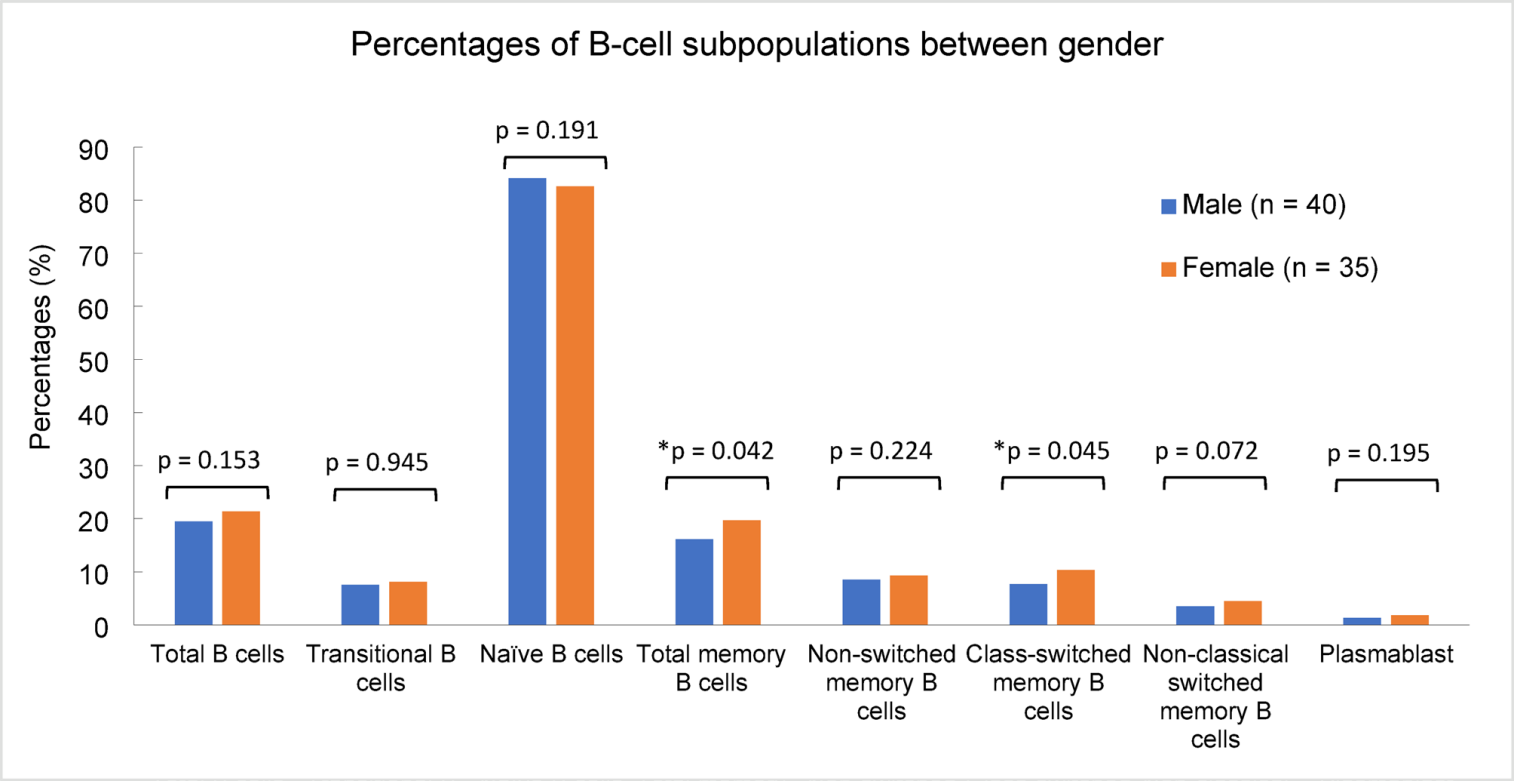
Gender-based frequency distribution of B-cell subpopulations in healthy Malaysian children aged 2–15 years. The bar chart depicting the absolute number of B-cell subpopulations between male (n = 40, blue bar) and female (n = 35, orange bar). Significantly lower total memory B cells (p = 0.042) and class-switched memory B cells (*p* = 0.045) were observed in males. *Indicates significant differences, *p* < 0.05.

Further analysis using Spearman’s rank-order correlation revealed a strong correlation between age and the absolute counts of total B cells, transitional B cells, naïve B cells, and non-switched memory B cells, with all four subsets demonstrating a statistically significant association (*p* < 0.001). Additionally, the Mann-Whitney U test was used to examine gender-related differences in B-cell subpopulations. The results indicated that female children had significantly higher total memory B cells frequency (*p* = 0.042) and class-switched memory B cells frequency (*p* = 0.045) compared to male children. However, no significant gender differences were observed in total B cells frequency (*p* = 0.153), total B cells absolute number (*p* = 0.734), transitional B cells frequency (*p* = 0.945), transitional B cells absolute number (*p* = 0.559), naïve B cells frequency (*p* = 0.191), naïve B cells absolute number (*p* = 0.932), total memory B cells absolute number (*p* = 0.124), non-switched memory B cells frequency (*p* = 0.224), non-switched memory B cells absolute number (*p* = 0.308), class-switched memory B cells absolute number (*p* = 0.053), non-classical switched memory B cells frequency (*p* = 0.072), non-classical switched memory B cells absolute number (*p* = 0.146), plasmablast frequency (*p* = 0.195), and plasmablast absolute number (*p* = 0.181), as illustrated in Fig. 3.

## Discussion

Various studies have validated the age-dependent distribution of B-cell subpopulations, especially among paediatric populations^12–17^. Each B cell subpopulation represents a distinct stage of the B-cell development, with full activation occurring upon antigen exposure. A reduction in the absolute number or frequency of specific B-cell subpopulations may raise concerns regarding underlying immunological conditions, including primary immunodeficiency^12,18–20^. While reference values for B-cell subsets have been established in several populations, no such values have been documented for Malaysia. To the best of our knowledge, this study is the first to establish reference values for paediatric B-cell subpopulations in Malaysian children below 15 years of age, specifically within the Clinical Immunology Centre at Hospital Sultan Abdul Aziz Shah, Universiti Putra Malaysia, Selangor.

In this study, we defined reference values for total B cells, transitional B cells, naïve B cells, total memory B cells, switched memory B cells, non-classical switched memory B cells, non-switched memory B cells, and plasmablasts in 75 healthy children aged 2 to 15 years. Although the physiological of each B-cell subset is not entirely elucidated, these subsets can be identified based on the significant surface marker CD19. As expected, a notable reduction in total B cells, marked by CD19 positivity (both in frequency and absolute number) was observed with increasing age during childhood. This trend has been previously reported and is known to continue as age increases^12^. Prior research has highlighted that ageing plays a key role in this decline, as bone marrow output capacity diminishes over time. Additionally, early B cell populations predominantly appear as transitional B cells in peripheral circulation before shifting into naïve B cells^12,21–23^. The observed reduction in both transitional and naïve B cells in this study mirrors the overall decline in total B cells. Importantly, the absolute number of total memory B cells and plasmablasts remained relatively stable over time (Tables 2 and 3). Taken together, these findings align with the previous research, suggesting that the reduction of total B cells may be attributed to declines in transitional and naïve B cell subpopulations^12^. Interestingly, severe reduction or complete absence of total B cells is commonly observed in patients with X-linked agammaglobulinemia (XLA). This is primarily caused by the genetic mutation in Bruton’s tyrosine kinase (BTK), which is responsible in mediating B cell development and maturation. The mutation in the BTK gene disrupts the progression from pro-B cells to pre-B cells, preventing the proper development of B cells and halting the hematopoietic stem cells from entering the B cell lineage. Thus, functional and antigen-responsive cells are unable to be generated^7,24–26^.

A continuous decline in the absolute number of non-switched memory B cells and non-classical switched memory B cells were also observed in this study, whereas the absolute count of switched memory B cells remained relatively stable across age groups. These findings are consistent with those reported by Duchamp et al. and Morbach et al.^12,27^. A decrease in switched memory B cells below the reference range has been suggested as a potential indicator for evaluating patients with suspected primary immunodeficiencies. Indeed, switched memory B cells have been incorporated into diagnostic criteria for common variable immunodeficiency (CVID) in the Freiburg, Paris and European classifications^18,28–30^. However, the cut-off values proposed by these classifications vary and are based on adult populations. This underscores the necessity of establishing age- and population-specific reference values for distinct B-cell subpopulations. Additionally, patient with either CD40 ligand or CD40 deficiency exhibit reduced memory B cells. Mutations in the CD40L and CD40 genes disrupt the CD40 activation pathway, resulting in defects in both class switch recombination (CSR) and somatic hypermutation. These defects have been categorized under combined T- and B-cell immunodeficiencies^7,25,31^.

Plasmablasts, characterized as CD19^+^CD27^+^CD38^+ bright^ B cells, are typically present in trace amounts within peripheral blood. Some researchers have stated that plasmablasts rarely exceed 5% of total B cells^12^. In our study, both the proportion and absolute number of plasmablasts were less than 2% and 2 × 10^6^ /L, respectively. Notably, plasmablast remained consistent across all age groups. It is crucial to recognize that the expansion of plasmablasts in the peripheral blood may indicate autoimmune rheumatic conditions, such as systematic lupus erythematous, Sjögren’s disease, and rheumatoid arthritis^32–36^. Therefore, establishing population-specific reference range could help define precise cut-off values for detecting autoimmune diseases.

Numerous studies have reported reference values for B-cell subpopulations derived from various regions and populations which include larger sample sizes, younger age groups, and more detailed phenotypic dissection of B-cell subsets. However, despite the availability of these datasets, comparative evaluation of regional variability in B-cell reference values remains limited mainly due to differences in age stratification, flow cytometry panels, and gating strategies. Generally, our findings demonstrate overall age-related trends in B-cell subpopulation distribution that are broadly consistent with French population^27^, Spanish population^15^, German population^12^, Mexican population^16^, and Swiss population^37^. Our data therefore provides an important regional reference framework for Malaysian children and may serve as a basis for contextual comparison in the future.

Interestingly, our study revealed gender-based differences in B-cell subpopulation distribution. Female children had a significantly higher frequency of total memory B cells and class-switched memory B cells compared to their male counterparts. This suggests that, in addition to age-related factors, gender also influences B cell subset distribution and immune responses. Two potential explanations for this phenomenon include the influence of sex hormones, such as oestrogen and testosterone, and the role of X chromosome in regulating immune-related genes^38,39^. Previous research has reported that oestrogen stimulates B cells proliferation, whereas testosterone delays this process^38,40^. Additionally, the X chromosome plays an important role in immune function, as it contains coding regions for genes involved in immune responses, including toll-like receptors, cytokine receptors, and transcription factors^38,41^. Consequently, gender should be considered when assessing immunological evaluations in young patients.

Despite the strengths of this study, several limitations should be acknowledged. The main limitation is the small sample size. This small sample size can be reflected in the statistical power, potentially compromising both the sensitivity and the specificity of the analysis. Primarily, it may cause difficulty in replicating results and increase the likelihood of false negative results, the type Ⅱ error^42,43^. According to the Clinical and Laboratory Standards Institute (CLSI), a minimum sample size of 120 subjects is required to establish reference intervals^44^. For studies involving multiple variables, such as in this study, at least 120 subjects per variable would be ideal. Although we were only able to recruit a small cohort of 75 participants, this study serves as an initial platform for determining reference values within our laboratory. However, these findings may not be generalizable to the entire Malaysian population. Future studies with larger cohorts are necessary to establish more comprehensive population-specific reference values. Another limitation of this study is the restricted age range of participants. Since our focus was on children aged 2 years to 15 years, the reference values do not account for B cell subpopulation variations in early infancy. Previous research suggests that significant fluctuations in B cell distribution frequencies and absolute counts occur within the first five years of life. Expanding the age range in future studies would allow for more thorough characterization of B-cell distribution across different developmental stages within the Malaysian population. Indeed, differences in vaccination schedules, environmental exposures, and regional infectious disease burdens may contribute to variability in B-cell subpopulation reference values across populations and warrant further investigation in future studies.

In conclusion, the characterization of B-cell subpopulations is crucial for evaluating primary immunodeficiencies. This study provides age-specific reference values for B cell subsets in a paediatric population, which may serve as a valuable guideline for diagnosing children with suspected immunodeficiency. Additionally, our findings highlight the potential impact of both age and gender on B cell distribution, emphasizing the need for further research in this area. Future studies should investigate whether variations in B cell subset differentiation and expansion are influenced by population-specific or environment factors.

## Data Availability

All data generated during this study are included in this published article. Raw datasets are available from the corresponding author upon reasonable request.
